# *Clonorchis sinensis* excretory/secretory products promote the secretion of TNF-alpha in the mouse intrahepatic biliary epithelial cells via Toll-like receptor 4

**DOI:** 10.1186/s13071-015-1171-0

**Published:** 2015-10-24

**Authors:** Chao Yan, Yan-Hong Wang, Qian Yu, Xiao-Dan Cheng, Bei-Bei Zhang, Bo Li, Bo Zhang, Ren-Xian Tang, Kui-Yang Zheng

**Affiliations:** Department of Pathogenic Biology and Immunology, Laboratory of Infection and Immunity, Xuzhou Medical College, Xuzhou 221004, Jiangsu Province, People’s Republic of China

**Keywords:** Toll-like receptor 4, *Clonorchis sinensis*, Biliary epithelial cells, Excretory/secretory products, Interleukin (IL)-6, Tumor necrosis factor (TNF)-α

## Abstract

**Background:**

Toll-like receptor 4 (TLR4), as one of the most important pathogen pattern recognitions (PPRs) plays a central role in elicitation of innate immunity and mediation of adaptive responses against foreign antigens. However, little is known of the roles of TLR4 in the immune responses of biliary epithelial cells (BECs) induced by *Clonorchis sinensis*, a parasite of significance in human health.

**Methods:**

In the present study, the primary mouse intrahepatic biliary epithelial cells (MIBECs) were pre-treated with TLR4 inhibitor peptide or control peptide and then stimulated by excretory/secretory products (ESP) of *C. sinensis*, respectively. The expressions of TLR4 and relative cytokines were determined using western blot and a bead-based analytic detection system, respectively.

**Results:**

The results showed that ESP of *C. sinensis* significantly increased the expression of TLR4 which promoted the expression of MyD88 and NF-κB in BECs; the levels of TNF-α but not IL-6 from MIBECs stimulated by ESP alone were also considerably increased, compared with the group of the medium stimulated. However, the concentration of TNF-α was significantly decreased when MIBECs were pre-treated with TLR4 inhibitor. In addition, ESP could depress the level of IL-6 in MIBECs which was elevated by LPS.

**Conclusions:**

Our data for the first time demonstrate that ESP of *C. sinensis* can potently induce secretion of pro-inflammatory cytokines via TLR4 in MIBECs, which suggests that TLR4 plays an important role in host defenses against *C. sinensis* and the pathogenesis of clonorchiasis.

## Background

Clonorchiasis, caused by *Clonorchis sinensis* is an important food-borne zoonosis which is widely prevalent in Eastern Asia including China, Korea, eastern Russia and Vietnam [1, 2]. It was estimated that about 15 million people were globally infected by *C. sinensis* while 12.5 million people were distributed in China [3, 4]. Humans and other piscivorous mammals such as cats and dogs get infected mainly via ingestion of the raw or under-cooked fresh fish that contain metacercariae of *C. sinensis*. After being swallowed, the juvenile worms are released upon the stimulation of digestive juices, and then move to biliary tract, where the worms inhabit and develop into adults. Infection with *C. sinensis* can lead to severe cholangeitis, cholecystitis, hepatic fibrosis and cirrhosis. Importantly, *C. sinensis* infection is closely related with cholangiocarcinoma in humans, although the mechanism of this remains unknown [4–6].

Cholangiocytes, also called biliary epithelial cells (BECs) lining in the bile ducts constitute approximate 4 % ~ 5 % of the total population of hepatic cells and are characterized by secretion of bile into the duodenum [7]. Accumulating studies demonstrated that cholangiocytes also play important roles in the hepatobiliary immunity since they provide the first line of fighting against foreign microbes in the biliary system. On the one hand, BECs acting as antigen presenting cells (APC) secrete cytokines/chemokines (eg. IL-6) and express a group of adhesion/co-stimulatory molecules, such as major histocompatibility (MHC) class II, intercellular adhesion molecule 1 (ICAM-1) and B7-H1, by which the cholangiocytes are capable of interaction with other lymphocytes and induce adaptive immune responses [8–10]; on the other hand, BECs are able to release human β-defensins (hBDs), Mx proteins and secretory immunoglobulin A (sIgA) which are involved in mucosal immunity and play a crucial role in host defense against microbial infection [11].

Toll-like receptor (TLR) is one of most important pathogen pattern recognitions (PPRs) which motivate intracellular signaling cascades via the nuclear-factor κB (NF-κB), caspase-dependent and mitogen-activated protein kinase (MAPK) signaling pathways to tirgger host’s immune responses [12]. Similar to other epithelium, BECs also express multiple Toll-like receptors and mediate inflammatory responses of host to fight against foreign antigens or microbes [13–16]. For example, upon stimulation of LPS, human BECs have the ability to secrete interleukin (IL)-6, IL-8 and tumor necrosis factor-α (TNF-α) through TLR4-NF-κB and TLR4-MAPK signaling pathways [17]; the expression of hBD2 and nitric oxide (NO) were upregulated in a human cholangiocyte cell line infected by *Cryptosporidium parvum* in a TLR2/TLR4-NF-κB dependent manner and TLR4 could significantly decrease the worm’s burden of *C. parvum* in a mouse model of biliary cryptosporidiosis [18–20]. Ninlawan et al. showed that the excretory/secretory products (ESP) of *Opisthorchis viverrini* could increase the expression of TLR4 in a MyD88-independent manner and induce the innate mucosal immunity against *O. viverrini* infection [21]. However, as the first line against *C. sinensis* infection, the mechanism by which the inflammatory responses of the cholangiocytes caused by *C. sinensis* infection remains unknown. In view of this background, the aim of the present study was to investigate the possible roles of TLR4 in inflammatory responses of a primary mouse intrahepatic biliary epithelial cell (MIBEC) stimulated by ESP of *C. sinensis*.

## Methods

### Animals and parasites

Female BALB/c mice (6 ~ 8 weeks, 20 ± 2 g) were purchased from Shanghai Laboratory Animal Co, Ltd (SLAC, Shanghai, China). The mice were housed in an air-conditioned room at 24 °C with a 12 h dark/light cycle and permitted free access to standard laboratory food and water.

The metacercariae of *C. sinensis* were obtained as described elsewhere [22]. In brief, the fish were minced by electric blender and digested with artificial gastric juice, a solution of 0.7 % pepsin A and 0.1 % HCl, at 37 °C in a shaking water bath for 12 h. The digested mixture was filtrated through a series of sieves with the mesh size of 1000 μm, 300 μm and 106 μm. Then the pellet was sedimented in phosphate buffered solution (PBS) in a sedimentation jar until the supernatant was clear. *C. sinensis* metacercariae were identified, collected under a dissecting microscope, and stored in PBS at 4 °C until use.

The New Zealand white rabbits were infected with approximate 800 *C. sinenis* metacercariae by intragastic intubation. The adult worms were collected on 56 day post-infection. The fresh worms were washed three times in sterile PBS containing penicillin (200 U/ml) and streptomycin (200 μg/ml) to remove any debris and residual blood. After washing thoroughly, the viable worms were collected for preparation of ESP.

### Ethical approval

All animal experimental procedures were reviewed and approved by the Animal Care and Use Committee of Xuzhou Medical College. The main procedures were compiled according to the guidelines of the National Laboratory Animal Center.

### Preparation of *C. sinensis* ESP

*C. sinensis* ESP were prepared as previously described with a minor modification [21]. Briefly, the fresh worms were cultured in sterile PBS with penicillin (100 U/ml), streptomycin (100 μg/ml) and protease inhibitors (0.1 mM Phenylmethanesulfonyl fluoride, PMSF). The worms were maintained in vitro at 37 °C up to 12 hours. The cultured fluid was collected and centrifuged at 10, 000 g for 10 min and the clear supernatant was pooled, concentrated and filtered through a 0.2 μm membrane, then they were aliquoted and stored at −80 °C . The concentration of endotoxin in ESP solution was detected using LAL Chromogenic Endotoxin Quantization kit (the detection limit is 0.005 Endotoxin Unit/ml, Zhanjiang A&C Biological Ltd, GuangDong, China). The ESP that contained no more than 0.005 EU/ml endotoxin were further used for stimulation assays. ESP concentration was measured by bicinchoninic acid protein determination method provided by a commercial available kit (Beyotime Biotechnology, Beijing, China).

### Isolation and identification of MIBECs

MIBECs with viability >90 % (trypan blue exclusion) were isolated from the liver of the healthy mice as described elsewhere with some modifications [23]. Briefly, the mouse was anaesthetized and portal vein was perfused with type IV collagenase, subsequently intrahepatic bile duct was removed and digested with DNase I, pronase E and type IV collagenase [24]. The small and uniform cells were cultured in Dulbecco’s Modified Eagle’s Medium/F12 medium containing 10 % heat-inactivated fetal bovine serum (Invitrogen, Carlsbad, CA, USA), 10 ng/ml epidermal growth factor (PeproTech, USA), 10 ng/ml hepatocyte growth factor (PeproTech, USA), insulin-transferrin-selenium (Gibco®, Life Tech, USA), 100 U/mL of penicillin, and 100 μg/mL streptomycin in a humidified atmosphere of 5 % CO_2_ at 37 °C. After cells attached to the plastic culture plate pre-treated with I type rat tail collagen, the purity and identification of cholangiocyte preparations were assessed by immunofluorescence assay of cytokeratin 19 (CK19).

### Culture and stimulation of MIBECs

After 5 days culture, MIBECs were seeded at a density of 1 × 10^5^ cells per well for various purposes. For ESP stimulation, MIBECs were incubated with *C. sinensis* ESP at 10 ~ 40 μg/ml for up to 48 h depending on different purposes. For TLR4 blocking assays, MIBECs were pre-treated with 10 μM TLR4 inhibitory peptide (amino acid sequence: KYSFKLILAEY) or control peptide (amino acid sequence: RNTISGNIYSA) for 2 hours prior to stimulation as described [25]. These TLR4 peptide inhibitor sets were commercially available (Novus Biologicals, Littleton, CO, USA). 1, 000 ng/ml LPS, 20 μg/ml ESP, or both and the medium (negative control) were added to stimulate with MIBECs for 48 h. After stimulation for 48 h, MIBECs were harvested for real time-PCR and western blot assays. Then cell supernatants were collected for the determination of the concentrations of TNF-α and IL-6.

### Immunofluorescence assay

After 5 days culture, MIBECs were fixed with methanol for 20 min at −20 °C, and then were permeated for 5 min by 0.3 % Tritonx-100 after washing three times. Subsequently, cells were incubated with blocking buffer containing 5 % FBS for 30 min at room temperature and then incubated with 5 μg/ml anti-mouse CK19 (Cell Signaling biotech Co, Ltd, MA, USA) and then incubated at 4 °C overnight. Cells were co-stained with 4’, 6-diamidino-2-phenylindole (DAPI, 1 g/ml) to visualize the nuclei. Stained cells were mounted with fluorescent mounting medium (Dako Cytomation) and assessed by a conventional fluorescent microscopy (Olympus BX51/DP71, Japan). The exposure time for FITC and DAPI signals was 2 seconds and 0.08 seconds, respectively. Negative controls for all assays were included in each staining.

### RNA isolation and quantitative real-time PCR

RNA was extracted from primary BEC cells using TRIzol reagent (Life Technologies, CA, USA), and cDNA was synthesized from the RNA using First Strand cDNA Synthesis kit (TIANGEN Biotech, Beijing, China). Following reverse transcription, cDNA was amplified using LightCycler® FastStart DNA Master (Roche Applied Science, Penzberg, Germany) with gene-specific primers designed to amplify a portion of the coding sequences. Quantitative PCR analyses were performed in a LightCycler® 480IIdetection system (Roche Applied Science, Penzberg, Germany) under the following thermal cycler conditions: one cycle of 5 min denaturation at 95 °C, and then 30 s at 95 °C, 30 s at 60 °C, and 30 s at 72 °C for 40 cycles using the primers as follows: *Tlr4*: Forward 5′-TGA CAG GAA ACC CTA TCC AGA GTT-3′; Reverse: 5′-TCT CCA CAG CCA CCA GAT TCT-3′; *Beta-actin*: Forward 5′-CGTGGGCCGCCCTAGGCACCA-3′; Reverse: 5′-TTGGCCTTAGGGTTCAGGGGGG-3′. All experiments were performed in triplicate and the Ct values were normalized to endogenous reference (beta-actin). The relative expression of TLR4 was indicated by comparative cycling threshold (Ct) normalized by β-actin with the 2^-△△Ct^ method.

### Western blotting

Total protein was extracted from MIBECs and analyzed with bicinchoninic acid protein concentration assay kit (Beyotime Biotech, Beijing, China). Sample protein was separated by electrophoresis in 12 % SDS-PAGE with a Bio-Rad electrophoresis system (Hercules, CA, USA). The primary antibodies (rabbit anti-TLR4, at 1 μg/ml, NF-κB p-65 at 1 μg/ml, MyD88 at 1 μg/ml antibody, Cell Signaling biotech Co, Ltd, MA, USA, 1:1000 dilutions) were incubated at 4 °C overnight. The secondary anti-bodies (anti-rabbit IgG, 1:5000 dilutions) was incubated for 1 h at room temperature. The membrane containing antibody-protein complexes were visualized with an enhanced chemiluminescence detection system on radiograph film (Bio-rad, Hercules, CA, USA). The bands were scanned and analyzed by the software Quantity ONE (Bio-rad, Hercules, CA, USA). The expression of protein in each sample was normalized by α-Tublin (Santa Cruz Biotechnology, CA, USA).

### Cytokine assay

The levels of TNF-α and IL-6 in the supernatant of the cultured MIBECs stimulated by *C. sinensis* ESP were measured by a bead-based analytic detection system (FlowCytomix Simplex kit, BD Biosciences, CA, USA) according to the manufacturer’s instruction. A standard curve for each cytokine was developed by mixing known quantities of recombinant mouse cytokines TNF-α and IL-6 in DMEM/F12 media for culture supernatant assays. The level of cytokine was determined by flow cytometry (Beckman Coulter Cytomics™ FC500) equipped with CXP software, and data were analyzed using FlowCytomix Pro 2.2 software. The sensitivities of the assays were 0.5 pg/mL for TNF-α and 1.2 pg/mL for IL-6, respectively.

### Statistical analysis

All values are expressed as mean ± SE. Means of groups were compared with the Student’s t test (unpaired) or ANOVA test when appropriate. *P <* 0.05 was considered statistically significant.

## Results and discussion

**Fig. 1:**
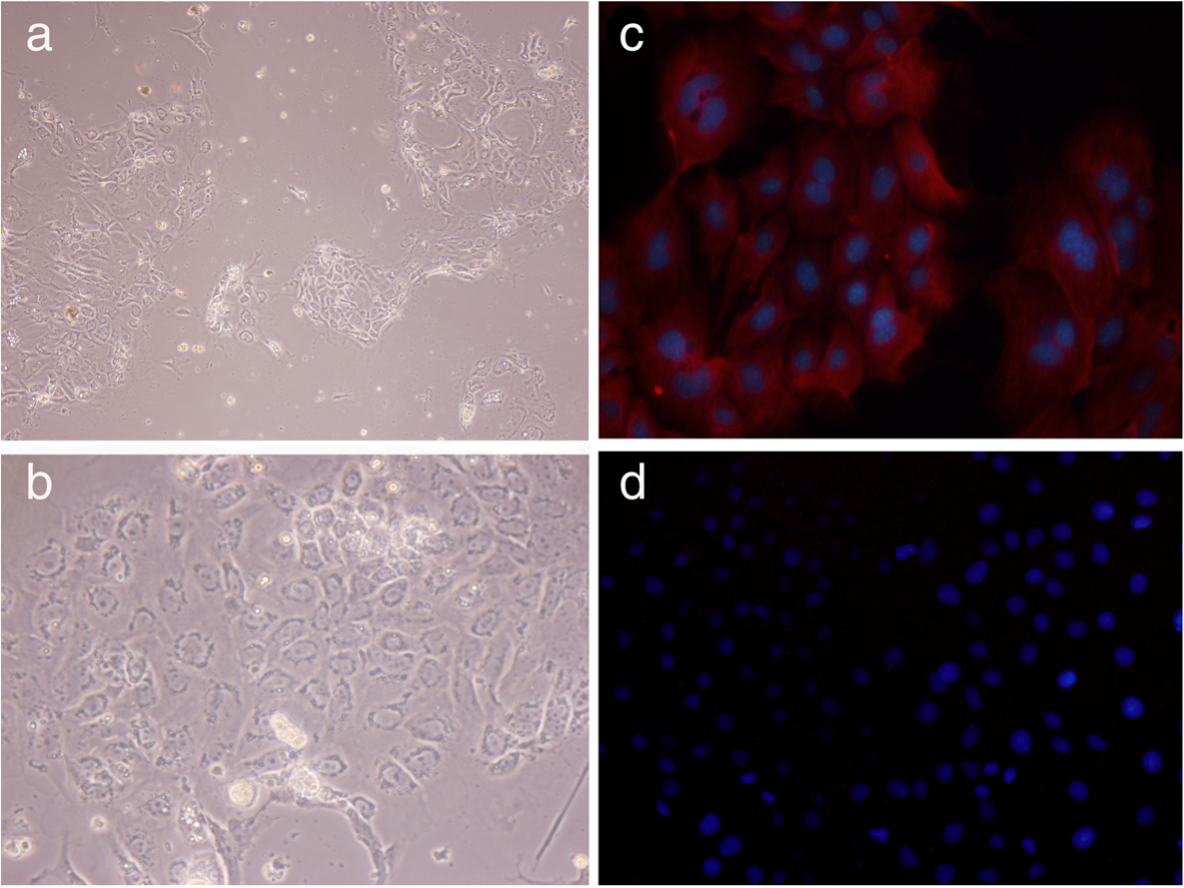
Characterization of primary mouse intrahepatic biliary epithelial cells (MIBECs) by immunofluorescence staining. **a** (200 × magnification) & (**b**) (400 × magnification). MIBECs were photographed under light field. **c** MIBECs were co-stained with anti-mice cytokeratin 19 (CK19) antibody and 4’, 6-diamidino-2-phenylindole (DAPI, 400 × magnification); (**d**) control cells (LO2 cells) were co-stained with anti-CK19 and DAPI (400 × magnification)

**Fig. 2:**
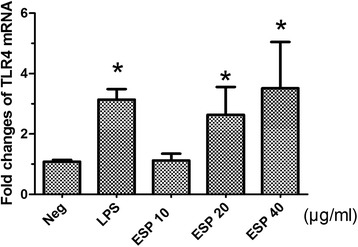
Relative expression of TLR4 in primary mouse intrahepatic biliary epithelial cells stimulated by different concentrations (10 μg/ml, 20 μg/ml and 40 μg/ml) of *Clonorchis sinensis* excretory/secretory products (ESP) using quantitative real-time PCR. Each value represents the mean ± SE of three or more independent experiments. *means significant differences (*P <* 0.05) were found, compared with negative group

As shown in Fig. 1, CK19, which is a biliary maker of epithelial cells, was specifically stained in the cytoplasm of the cultured cells, but the control cells (human normal hepatic line, LO2 cells) were negatively stained by anti-CK19 monoclonal antibody, suggesting the cholangiocytes were successfully isolated from the liver of mice and the purity of isolated cells was >95 %. As shown in Fig. 2, the level of the TLR4 mRNA was increased in MIBECs with the increase of the concentration of ESP between 10 μg/ml and 40 μg/ml, and ESP with the concentration of 20 μg/ml and 40 μg/ml could potently induce the significant expression of TLR4 mRNA, compared with the medium stimulated group (*P* < 0.05).

**Fig. 3:**
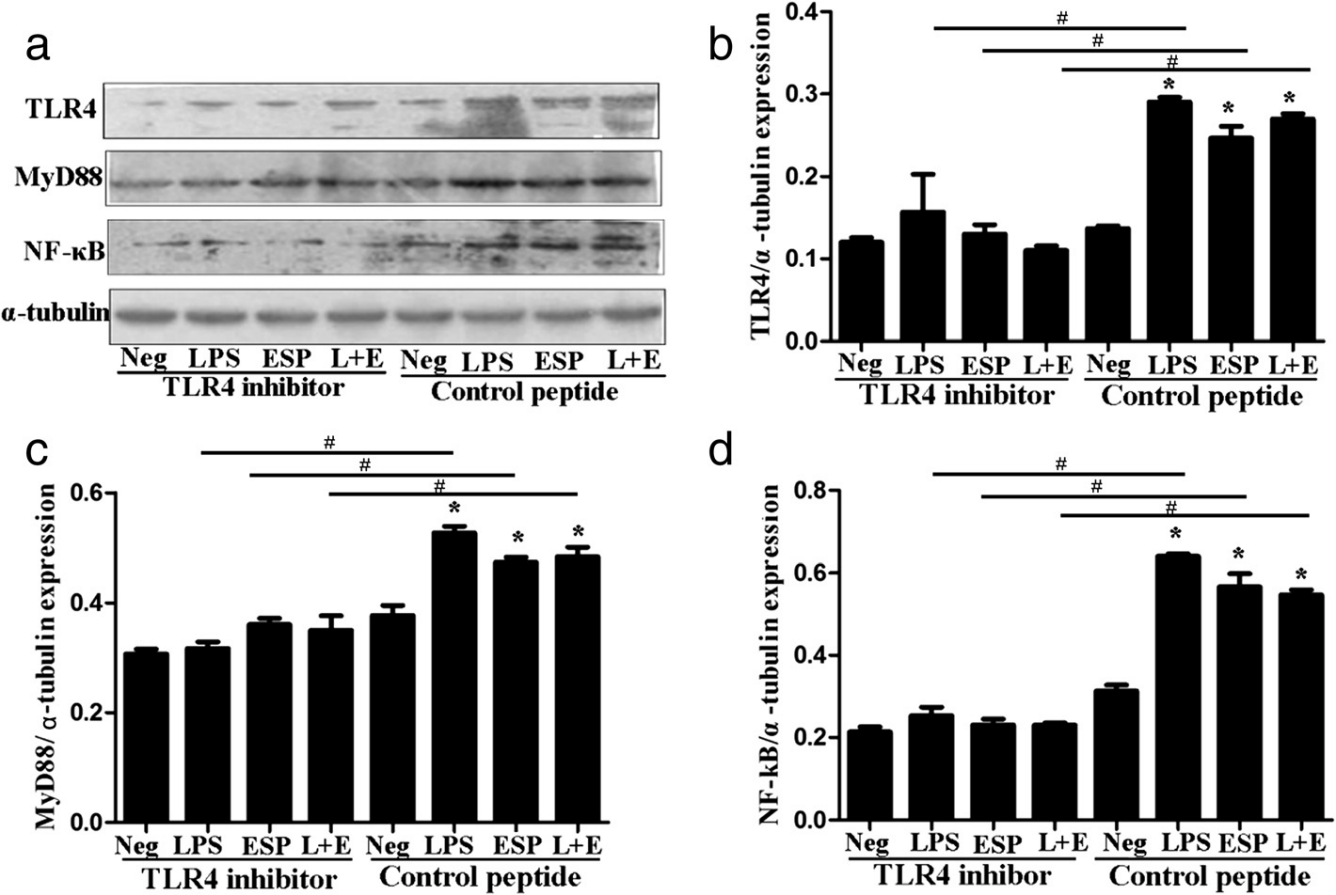
TLR4, MyD88 and NF-κB p65 protein expression in primary mouse intrahepatic biliary epithelial cells (MIBECs). MIBECs were pre-treated with control peptide group and TLR4 inhibitor (VIPER) group and stimulated by medium (negative control), LPS, excretory/secretory products of *C. sinensis* (ESP) and LPS + ESP (L + E) for 48 h, respectively. Each value represents the mean ± SE of three or more independent experiments. (**a**) The gel images of TLR4, MyD88 and NF-κB p65 showed by western blot .The relative expression of TLR4 (**b**), MyD88 (**c**) and NF-κB p65 (**d**) in each group. *represents significant differences (*P <* 0.05) were found, compared with the negative groups; #represents significant differences (*P* < 0.05) were found, compared with the indicated group

The results in our present study showed that LPS, ESP or both could potently increase the expression of TLR4, as well as the proteins of its downstream signaling pathways including the adapter protein MyD88 and transcription factor NF-κB (p65) in MIBECs exposed for 48 h (Fig. 3), suggesting that ESP from *C. sinensis* could potently promote the expression of the TLR4 and activation of TLR4 mediated intracellular signaling including MyD88 and NF-κB proteins. In addition, the results of the present study also showed that expressions of TLR4 and downstream signaling proteins including MyD88 and NF-κB p65 were significantly attenuated when a TLR4 inhibitor were pre-treated for 2 h, suggesting that TLR4 might be involved in the inflammatory immune response triggered *by C. sinensis* in the cholangiocytes.

**Fig. 4:**
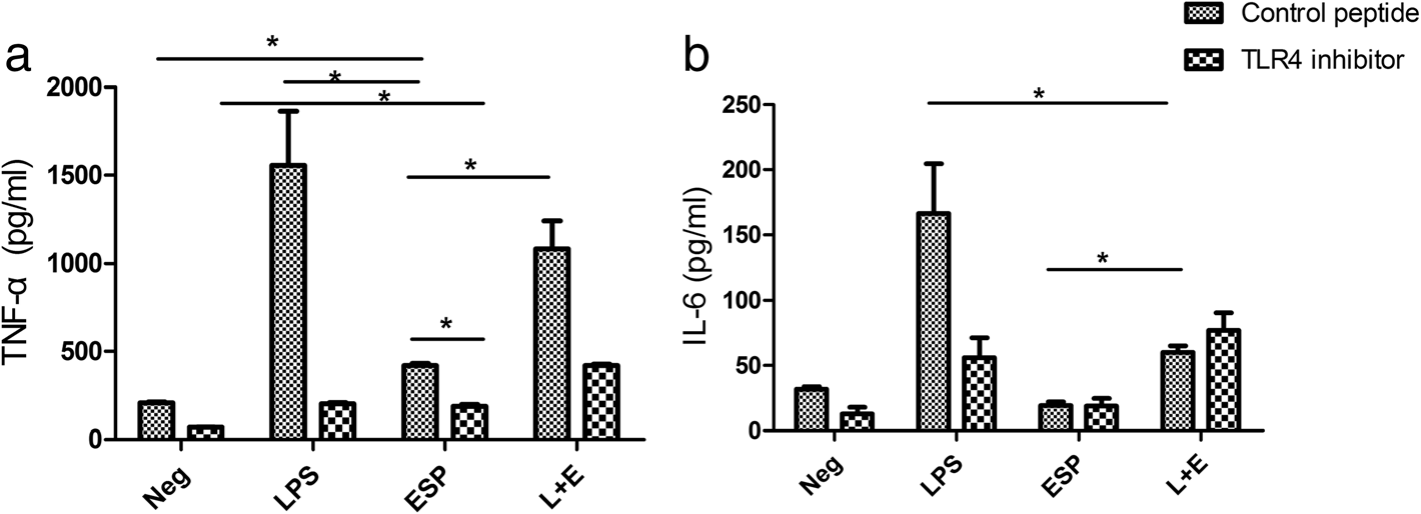
Cytokines levels of TNF-α and IL-6 in culture supernatants of primary mouse intrahepatic biliary epithelial cells stimulated by excretory/secretory products of *Clonorchis sinensis* (ESP) by bead-based flow cytometery. (**a**) The concentrations of TNF-α in medium (negative control), LPS and ESP, as well as LPS combined with ESP (L + E) group; (**b**) The concentrations of IL-6 in each group above. Each value represents the mean ± SE of three or more independent experiments. *represents significant differences (*P <* 0.05) were found, compared with the indicated group

For pro-inflammatory cytokines, the results showed that ESP alone could directly enhance the secretion of TNF-α but not IL-6 from MIBECs, compared with negative control group (Fig. 4a, *P* < 0.05). Moreover, the concentration of TNF-α from MIBECs stimulated by ESP alone was markedly decreased when TLR4 was blocked by a peptide -VIPER, compared with control peptide pre-treated MIBECs (Fig. 4a, *P* < 0.05). Interestingly, the concentration of TNF-α in the MIBECs stimulated by ESP alone was still increased when TLR4 was blocked, suggesting other signaling pathway may contribute to the production of TNF-α in addition to TLR4 signaling pathway. With regard to IL-6, surprisingly, ESP alone could not enhance the secretion of IL-6, compared with negative control (Fig. 4b, *P* > 0.05), and there were no statistical differences for the concentrations of IL-6 in MIBECs stimulated by ESP alone or ESP pulsed with LPS between TLR4 inhibitor pretreated groups and control peptide control groups (*P* > 0.05). However, the concentration of IL-6 were significantly decreased when MIBECs were co-stimulated by LPS and ESP, compared with LPS stimulated alone (Fig. 4b, *P* < 0.05), suggesting that ESP could potently depress the secretion of IL-6 from MIBECs partly by targeting at TLR4.

TLRs family is one of most important pathogen recognition molecules that mediate and shape the types of the inflammatory responses. In our previous study, we found that the expression of TLRs (TLR2 and TLR4) were significantly increased in a mouse model of clonorchiasis and the protein of TLR4 was strong stained in the mouse intrahepatic biliary epithelial cells in the mice infected with *C. sinensis* metacercariae on day of 28 post-infection [26]. However, the roles of TLR4 in mediation of immune responses during *C. sinensis* infection are still unknown. Actually, the expressions and roles of different TLRs and its downstream signaling pathway may be distinct due to various conditions, for example, Pinlaor et al. showed that TLR2 but not TLR4 was up-regulated in Raw 264.7 macrophage cells stimulated by *O. viverrini* somatic extracts, which subsequently triggered the production of nitric oxide synthase (iNOS) and cyclooxygenase-2 (COX-2) through NF-κB signaling [27]. Similar to our results, ESPs from *O. viverrini* could only increase the TLR4 among different TLRs expressed on the surface of the cholangiocytes and induce the activation of NF-κB in a MyD88-dependent manner [21]. Chen et al. demonstrated that the expressions of TLR2 and TLR4 were both up-regulated in a normal human biliary cell (H69) stimulated by *C. parvum* and the upregulations of TLR2 and TLR4 mediated human beta-defensin-2 expression to protect from microbe infection via activation of NF-κB [18]. Herein, the results of the present study demonstrated that the up-regulated TLR4 might play a role in the immune responses of *C. sinensis*.

Previous studies showed that ESP from *C. sinensis* could significantly promote hepatic fibrosis and were considered to be a carcinogenic factor of cholangiocarcinoma [28, 29]. However, the secretion of cytokine/chemokines in BECs, which might interact closely with *C. sinensis* as well as its ESP, is unknown and the roles of TLR4 in inflammatory responses in the BECs stimulated by ESP of *C. sinensis* remains to be covered. In the present study, we found that TNF-α but not IL-6 were significantly increased in the supernatant of cultured MIBECs stimulated by ESP of *C. sinensis* for 48 h, suggesting that BEC might play a crucial role in immunoregulation and immune responses to *C. sinensis* infection since TNF-α can potently regulate immune responses and induce nitric oxide, which lead to the parasites death and the healing of damaged tissues [30, 31]. Surprisingly, IL-6 secreted by MIBECs could not be significantly triggered by ESP of *C. sinensis*, however, when MIBECs co-stimulated by ESP and LPS, the levels of IL-6 in cultured supernatant of MIBECs were statistically decreased, compared with those stimulated by LPS alone (Fig. 4b), indicating that some components in ESP could negatively regulate the secretion of IL-6 in BECs against *C. sinensis* by targeting at TLR4. However, the results of the present study were not in agreement with other studies which showed a considerable augment of IL-6 levels in the BECs exposed to other foreign antigens such as *O. viverrini* ESP [21]. The mechanisms contributed to the difference should be further investigated.

## Conclusion

In conclusion, the present study, for the first time shows that ESP of *C. sinensis* can induce secretion of TNF-α but not IL-6 via TLR4 in vitro, suggesting that TLR4 which mediates pro-inflammatory responses in BECs serves as a primary component that may contribute to defenses of host against *C. sinensis* infection and the pathogenesis of clonorchiasis which is of significance for health of human and animal.
